# A Novel *CLN6* Variant Associated With Juvenile Neuronal Ceroid Lipofuscinosis in Patients With Absence of Visual Loss as a Presenting Feature

**DOI:** 10.3389/fgene.2021.746101

**Published:** 2021-11-19

**Authors:** Paschalis Nicolaou, George A. Tanteles, Christina Votsi, Eleni Zamba-Papanicolaou, Savvas S. Papacostas, Kyproula Christodoulou, Yiolanda-Panayiota Christou

**Affiliations:** ^1^ Department of Neurogenetics, The Cyprus Institute of Neurology and Genetics, Nicosia, Cyprus; ^2^ Cyprus School of Molecular Medicine, The Cyprus Institute of Neurology and Genetics, Nicosia, Cyprus; ^3^ Department of Clinical Genetics and Genomics, The Cyprus Institute of Neurology and Genetics, Nicosia, Cyprus; ^4^ Department of Neuroepidemiology, The Cyprus Institute of Neurology and Genetics, Nicosia, Cyprus; ^5^ Department of Neurobiology, The Cyprus Institute of Neurology and Genetics, Nicosia, Cyprus

**Keywords:** neuronal ceroid lipofuscinosis, batten disease, CNL6, next-generation sequencing, lysosomal storage disorders, *in silico* prediction

## Abstract

The neuronal ceroid lipofuscinoses (NCLs), also known as Batten disease, are a group of autosomal recessive lysosomal storage disorders that are characterized by neurodegeneration, progressive cognitive decline, motor impairment, ataxia, loss of vision, seizures, and premature death. To date, pathogenic variants in more than 13 genes have been associated with NCLs. *CLN6* encodes an endoplasmic reticulum non-glycosylated transmembrane protein, which is involved in lysosomal acidification. Mutations in *CLN6* cause late-infantile juvenile NCL (JNCL) adult-onset NCL, and Kufs disease. Members from two available families with JNCL were clinically evaluated, and samples were collected from consenting individuals. The molecular investigation was performed by whole-exome sequencing, Sanger sequencing, and family segregation analysis. Furthermore, *in silico* prediction analysis and structural modeling of the identified *CLN6* variants were performed. We report clinical and genetic findings of three patients from two Greek-Cypriot families (families 915 and 926) with JNCL. All patients were males, and the first symptoms appeared at the age of 6 years. The proband of family 926 presented with loss of motor abilities, ataxia, spasticity, seizure, and epilepsy. The proband of family 915 had ataxia, spasticity, dysarthria, dystonia, and intellectual disability. Both probands did not show initial signs of vision and/or hearing loss. Molecular analysis of family 926 revealed two *CLN6* biallelic variants: the novel, *de novo* p.Tyr295Cys and the known p.Arg136His variants. In family 915, both patients were homozygous for the p.Arg136His *CLN6* variant. Prediction analysis of the two *CLN6* variants characterized them as probably damaging and disease-causing. Structural modeling of the variants predicted that they probably cause protein structural differentiation. In conclusion, we describe two unrelated Cypriot families with JNCL. Both families had variants in the *CLN6* gene; however, they presented with slightly different symptoms, and notably none of the patients has loss of vision. *In silico* prediction and structural analyses indicate that both variants are most likely pathogenic.

## Background

The neuronal ceroid lipofuscinoses (NCLs), also known as Batten disease, are the most common autosomal recessive neurodegenerative diseases, characterized by the accumulation of auto-fluorescence lipopigments in various tissues and cell types. The prevalence of these groups of diseases is about 1:10,000 and 1:12,500 (Haltia, 2006; Moore et al., 2008). The main clinical features of the disease include retinopathy with visual loss, progressive epilepsy, dementia, ataxia, and motor and mental deterioration (Haltia, 2006; Cannelli et al., 2009; Sato et al., 2016). NCLs are clinically classified into four major types based on the age of onset of the disease: infantile (6–24 months), late-infantile (2–4 years), juvenile (5–10 years), and adult-onset (>18 years) (Mink et al., 2013). Based on associated genes, NCLs are currently classified into 14 types (Table 1) (Williams and Mole, 2012).

**TABLE 1:** NCL classification based on associated genes.

Locus name	Inher	Gene	Phenotype	Omim
CLN1	AR	*PPT1*	Infantile (INCL), late-infantile (LINCL), juvenile (JNCL), adult (ANCL), (Kufs disease)	* 600,432
CLN2	AR	*TPP1*	Late-infantile (LINCL), juvenile (JNCL)	* 607,998
CLN3	AR	*CLN3*	Juvenile (JNCL), adult (ANCL), (Kufs disease)	* 607,042
CLN4	AD	*DNAJC5*	Adult (ANCL), (Parry disease)	* 302,910
CLN5	AR	*CLN5*	Late-infantile (LINCL), adult (ANCL), (Kufs disease)	* 608,102
CLN6	AR	*CLN6*	Late-infantile (LINCL), juvenile (JNCL), adult (ANCL), (Kufs disease)	* 606,725
CLN7	AR	*MFSD8*	Late-infantile (LINCL)	* 611,124
CLN8	AR	*CLN8*	Late-infantile (LINCL), Northern epilepsy (NE)	* 607,837
CLN9	AR	Unknown	—	%609,055
CLN10	AR	*CTSD*	Congenital late-infantile (LINCL), adult (ANCL), (Kufs disease)	* 116,840
CLN11	AR	*GRN*	Adult (ANCL), (Kufs disease)	* 138,945
CLN12	AR	*ATP13A2*	Juvenile (JNCL)	* 610,513
CLN13	AR	*CTSF*	Adult (ANCL), (Kufs disease)	* 603,539
CLN14	AR	*KCTD7*	Infantile (INCL)	* 611,725

Note. NCL, neuronal ceroid lipofuscinosis.

NCL caused by biallelic variants in the *CLN6* gene usually presents in early to late childhood to juvenile, between 1.5 and 8 years of age, with slow motor degeneration, ataxia, loss of vision, seizures (epilepsy), and mental disabilities. Early death by 12–15 years of age is reported. Variants in *CLN6* may also lead to a rare adult-onset form of NCL in which the symptoms present in adulthood, typically after age 30 years. The typical symptoms include epilepsy, ataxia, dysarthria, progressive loss of intellectual function, and commonly no vision loss. Adults with this disorder do not usually survive more than 10 years after diagnosis (Kleine Holthaus et al., 2019). Seizure treatment in the context of NCL is challenging due to the complexity of the disease (Schulz et al., 2013). *CLN6* encodes an endoplasmic reticulum (ER) non-glycosylated membrane protein, which plays a role in lysosomal function. The NCL protein 6 contains seven transmembrane (TM) domains, an amino-terminal domain, a carboxyl-terminal domain, and six loops connecting the TM domains. The TM domains are TM1 to TM7 from the N-terminus to the C-terminus (Wheeler et al., 2002; Mole et al., 2004).

We hereby present two unrelated Greek-Cypriot families with juvenile NCL (JNCL). The clinical features of patients were different. The main clinical symptoms of family 926 patient include motor disabilities, ataxia, spasticity, dysarthria, seizures, and epilepsy, whereas patients of family 915 had ataxia, spasticity, dysarthria, dystonia, and intellectual disability with no seizure or epilepsy. Patients in family 915 were homozygous for a known pathogenic *CLN6* variant, whereas the patient of family 926 was compound heterozygous for the known and novel *de novo CLN6* variant. Furthermore, these patients did not have either vision or hearing loss as presenting features. *In silico* prediction analysis supports the likely pathogenicity of the two variants.

## Materials and Methods

### Subjects and Samples

**FIGURE 1:**
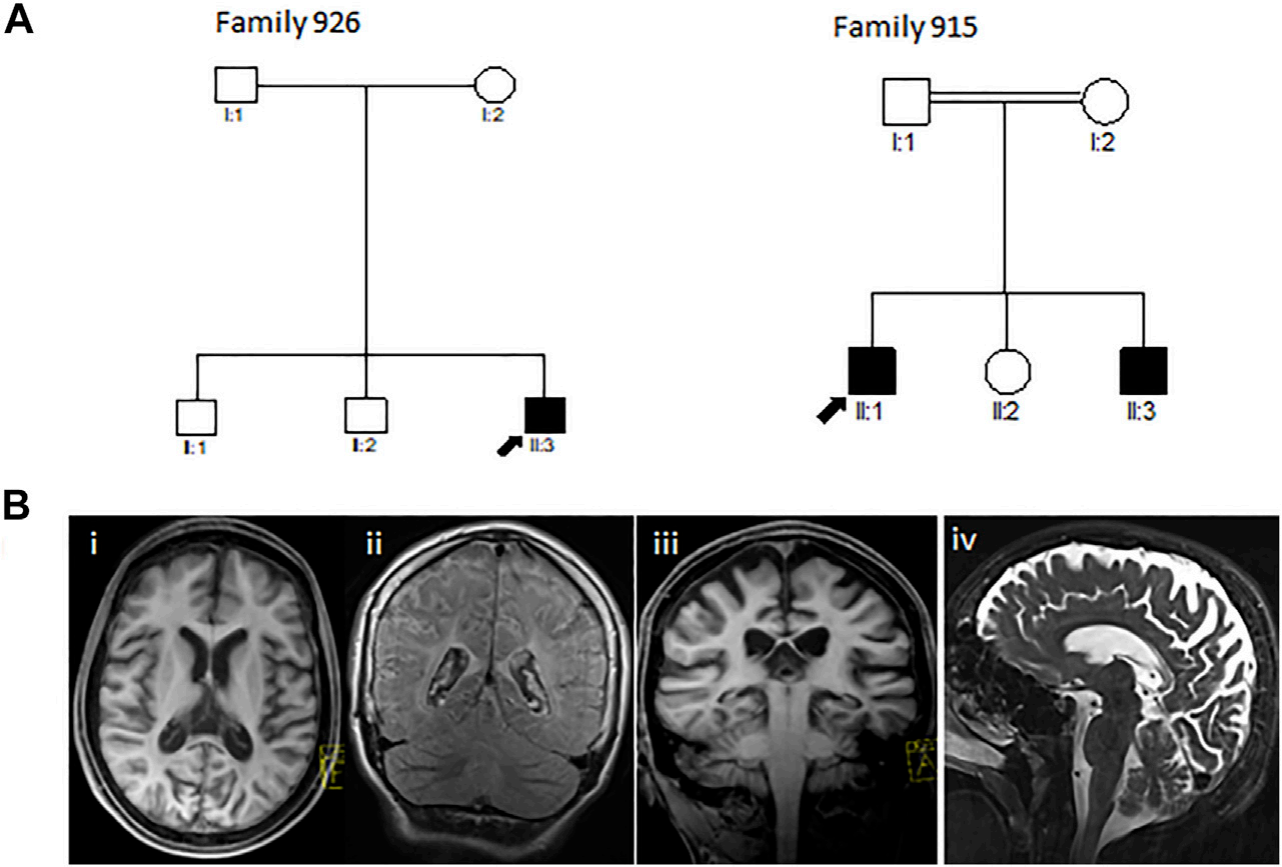
**(A)** Pedigrees of the reported Greek-Cypriot families (families 926 and 915). **(B)** Representative axial (i), coronal (ii, iii), and sagittal (iv) T1 and T2-weighted MRI images of the brain of the proband from family 926 aged 16 years demonstrating diffuse hypomyelination, thin corpus callosum, and cerebral and cerebellar atrophy.

Through this study, we investigated two Greek-Cypriot families. Family 926 was a three-generation family with one affected and six unaffected family members. Family 915 was a two-generation consanguineous family with two affected and three unaffected family members (Figure 1A).

All affected individuals were clinically evaluated in detail by the participating clinicians (CPY, EZ-P, SP, and GT), family history was obtained, and MRI brain scan and magnetic resonance spectroscopy (MRS) were performed. In addition, for family 926, cardiology and ophthalmological examination were performed. Blood samples were collected from consenting individuals, and genomic DNA was isolated using the Qiagen Gentra Puregene Blood Kit (Qiagen, Dusseldorf, Germany). Ethical approval was granted by the Cyprus National Bioethics Committee (EBKK/EΠ/2013/18, May 14, 2015).

### Variant Analysis

#### Whole-Exome Sequencing (WES)

Whole-exome sequencing (WES) was carried out on the DNA sample of family 915 proband (II:1) as previously described (Lambrianides et al., 2020). The SureSelect whole-exome enrichment (v4) kit (Agilent Technologies, Santa Clara, CA, USA) was used, and the sample was prepared according to Agilent’s Sure Select protocol version 1.2 (Agilent Technologies). Sequencing was performed on the Illumina HiSeq 2000 (Illumina, Inc., San Diego, CA, USA) using the TruSeq v3 all exons (Agilent Technologies) and the 100-bp paired-end read mode to a mean target coverage of 20×.

Data were analyzed using an in-house-developed exome analysis pipeline. Reads were aligned to the GRCh37 1000 Genomes reference using bwa 0.6.2. Local realignment around indels was performed with the Genome Analysis Toolkit (GATK) version 1.6. Optical and PCR duplicates were excluded using the PICARD tool Mark Duplicates version 1.89. HiSeq base quality scores were recalibrated using GATK Table Recalibration, and variants were called with GATK Unified Genotyper. Variants were annotated with gene and gene function data from Ensembl and known variants from dbSNP (release 135). Initially, WES *in silico* NCL gene panel analysis was performed. The NCL panel included *ATP13A2*, *CLN3*, *CLN5*, *CLN6*, *CLN8*, *CTSD*, *DNAJC5*, *GRN*, *KCTD7*, *MFSD8*, *PPT1*, *PPT2*, *TPP1*, and *CTSF* genes. Candidate variants were then filtered based on frequency (<0.5% in the 1000 Genomes, ExAC, and Exome Variant Server (EVS)), inheritance of the disease, effect on protein sequence, and deleteriousness predictions of the variants. Filtering of the variants was further facilitated by comparing the resulting proband data with 76 control Cypriot exome data (152 chromosomes).

#### Sanger Sequencing

Genomic DNA sequencing of the candidate variants in CLN6 was performed as previously described (Nicolaou et al., 2008). PCR primers were designed using the Primer3 program (Whitehead Institute for Biomedical Research, Cambridge, MA, USA) and are available upon request. PCR amplification of candidate genes was performed using standard methods, and PCR products were purified with ExoSAP-IT for PCR product clean-up (Affymetrix, Santa Clara, CA, USA). Relevant PCR products were covered by both forward and reverse strand sequencing using the BigDye Terminator v1.1 Cycle Sequencing Kit (Applied Biosystems, Carlsbad, CA, USA) and the ABI 3130xl Genetic Analyzer (Applied Biosystems) according to the manufacturer’s protocol. Sequence traces were automatically compared with the normal gene sequences as listed in the GenBank database, using ABI SeqScape software (Applied Biosystems).

All available family members were analyzed for the identified variants to obtain direct evidence of the variant and its co-segregation with the disease in the family. Furthermore, 150 Cypriot control samples were checked for the identified variants.

#### Microsatellite Analysis

Microsatellite analysis was performed to confirm paternity in the *de novo* case as previously described (Nicolaou et al., 2013). Short tandem repeats were PCR amplified using fluorescently labelled forward primers for the following markers: D2S2379, D4S395, D5S639, D11S4098, D16S3098, and DXS8069. Detection was achieved using an ABI genetic analyzer (AB Applied Biosystems 3130xl), and analysis was performed using the Genemapper software (Applied Biosystems).

#### *In silico* Prediction Analysis and Structural Modeling of the *CLN6* Variants

*In silico* prediction analysis was performed in order to predict the effect of the variants, using the Polymorphism Phenotyping v2 (PolyPhen-2) (http://genetics.bwh.harvard. edu/pph2/dbsearch.shtml), the PROVEAN (http://provean.jcvi.org/index.php), the SIFT (http://sift.jcvi.org/www/SIFT_enst_submit.html), and the MutationTaster (http://www.mutationtaster.org/index.html) prediction tools. In addition, the VarCards *in silico* variant prediction tool was used (http://varcards. biols.ac.cn) (Li et al., 2018).

The protein structure prediction program Raptor-X (Kallberg et al., 2012) was adopted to predict the structure of the wild-type (WT) and mutated domains of *CLN6*. As a template for the prediction of the protein structures, Protein Data Bank (PDB) ID: *2yevA*, *3wajA*, *5amrA*, *3ayfA*, *5i6xA*, and *3rkoB* with alignment scores 75, 76, 73, 76, 70, and 80, respectively, were used. Predicted structures of the WT and variant CLN6 were visualized using the PyMOL software (The PyMOL Molecular Graphics System, Version 1.1 Schrödinger, LLC).

## Results

### Clinical Features

#### Family 926

The proband (III3) of family 926 (Figure 1A) developed significant hyperbilirubinemia soon after birth, for which he required an exchange transfusion. He acquired his milestones normally up to the age of 6 years, being able to read and write by which time he started developing learning issues with reduced attentiveness, poor concentration, and mild gait ataxia. A neuropsychological evaluation during that time showed a below-average IQ and attention deficit hyperactivity disorder (ADHD). At the age of 10 years, he presented with a tonic seizure that occurred during sleep. A second episode followed a few months later associated with focal jerking of the right lower limb and secondary generalization. He was investigated with electroencephalography (EEG) with evidence in keeping with rolandic epilepsy. Progressively, his condition deteriorated, and the patient presented with further loss of motor abilities, spastic gait, and unsteadiness along with cognitive regression and intractable myoclonic epileptic seizures. A brain MRI at 11 years revealed a degree of hypomyelination, and follow-up MRI 4 years later showed some additional volume loss and hypogenesis of the corpus callosum (Figure 1B). MRS demonstrated gray matter disease with atrophy of the vital neuroaxonal tissue. By the age of 17 years, the patient was wheelchair-bound and was able to perform only a few steps with support. He continued to exhibit epileptic episodes almost daily, which occurred mainly during sleep. Hair loss was also noted. There were initially no signs of hearing or visual loss. By the age of 18 years, the patient deteriorated further, developing dysphagia, and a gastrostomy tube was inserted. Ophthalmological assessment at the time showed evidence of reduced visual activity with no additional abnormalities. Further investigation with visual electrophysiology included normal visual evoked potentials and unobtainable pattern electroretinography responses bilaterally.

Investigation with plasma amino acids, organic acids, lactate, acylcarnitines, glycosaminoglycans (GAGs), oligosaccharides, plasma folate glucose, copper, vitamin B12, very long chain fatty acids (VLCFAs), serum immunoglobulins, alpha-fetoprotein, transferrin isoforms, urate, ceruloplasmin, purines screen, vacuolated lymphocytes, white cell enzymes and ubiquinone, organic acids, creatine kinase (CK), and acylcarnitines were in the normal ranges. Liver function tests (LFTs) have shown an elevation particularly of his alanine transaminase (SGPT). Cardiology assessment did not show any abnormalities. An abdominal ultrasound scan and gastroscopy did not reveal any pathology.

The patient received several combinations of antiepileptic medication with only partial improvement of his seizures, including phenobarbitone, oxcarbazepine, lamotrigine, levetiracetam, zonisamide, and lacosamide. He continued with intractable epileptic seizures, which significantly improved on treatment with valproic acid, but due to intolerance, this was discontinued. At the age of 18 years, he was admitted to the intensive care unit (ICU) due to an episode of status epilepticus, where he was intubated and had a tracheostomy. During the following weeks, there was no improvement, and any attempt to wake the patient resulted in multiple epileptic seizures or myoclonic crescendo. On the eighth week of his admission, off-label use of a combination of cannabidiol and tetrahydrocannabinol was given as palliative treatment followed by remarkable improvement of the patient’s seizures, and 2 weeks later, he was able to come out of sedation and commence physiotherapy.

The patient is currently 28 years old. He is mostly bedridden with spastic tetraparesis and almost anarthric. He is fed via a gastrostomy tube and continues with occasional seizure episodes.

#### Family 915

The proband (II1) of consanguineous family 915 (Figure 1A) was examined for the first time by the neurologist at the age of 6 years. The patient initially had signs of progressive spinocerebellar ataxia with retained tendon reflexes. The first symptoms also appeared in his younger brother at the age of 7 years. The main clinical characteristics of the affected family members were gait ataxia, spasticity, dysarthria, and dystonia with no initial evidence of visual failure. There was also no initial history of seizures or hearing impairment. They had intellectual disability, and their brain MRI showed cerebellar atrophy (MRI not shown). MRI from the very early stages of the disease did not show evidence of thin corpus callosum (TCC) or white matter lesions (WML) (Table 2). The family was subsequently lost to follow-up.

**TABLE 2:** The main clinical characteristics of patients from the two families included in this study.

Fam#	AOO	GA	SP	D	Ext	PNP	PEO	Ret/OΑ	Dem/Ps	LD/ID	Ep	CA	TCC	WML
F-926	6	+	+	+	?	?	—	—	+/?	1/2 ID?	+	+	?	+?
F-915	6	+	+	+	Dys	—	—	—	—	1/2 ID	-	+	—	—

Note. AOO, age of onset; GA, gait ataxia; SP, spasticity; D, dysarthria; Ext, extrapyramidal symptoms; T, tremor; Dys, dystonia; PNP, peripheral neuropathy; PEO, progressive external ophthalmoplegia; Ret, retinopathy; OA, optic atrophy; Dem, dementia; Ps, psychosis; LD, learning disability; ID, intellectual disability; Ep, epilepsy; CA, cerebellar atrophy; TCC, thin corpus callosum; WML, white matter lesions.

### Molecular Analysis

#### Family 915

Molecular analyses of the proband of family 915 for *SCA1*, *SCA2*, *SCA3*, *SCA6*, *SCA7*, *SCA8*, *SCA10*, *SCA12*, *SCA17*, *DRPLA*, *APTX*, *FRDA*, and *HD* were performed; and no disease-associated variant was found in these genes. The WES of the proband generated 76 million reads of 100-bp paired-end read sequences. More than 99.28% of all reads were successfully mapped to the reference genome, and a read depth >20× for 93.65% of the bases was obtained.

WES *in silico* NCL gene panel analysis revealed 19 genetic variants, and further filtering analysis reduced the number of candidate variants to five (*CLN6* c.407G > A, *GRN* c.421G > A, *CTSD* c.173C > T and c.465T > C, and *KCTD7* c.654C > T). Variants that were frequent in the WESs of Cypriot controls were excluded, and thus, the number of candidate variants was reduced to two (*CLN6* c.407G > A, p.Arg136His and the *GRN* c.421G > A, p.Val141Ile). Segregation analysis in the family with Sanger sequencing confirmed the *CLN6* homozygous variant c.407G > A, p.Arg136His in exon 4.

The above variant was absent from 150 Cypriot control samples.

#### Family 926

Karyotype and array comparative genomic hybridization (CGH) analysis of the proband of family 926 did not show any abnormalities. Furthermore, molecular analyses for fragile X syndrome, *SCA2*, *SCA3*, *SCA6*, *SCA7*, *SCA8*, *SCA12*, *SCA17*, *DRPLA*, and *CLN3* was performed and did not reveal any disease-associated variant in these genes.

**FIGURE 2:**
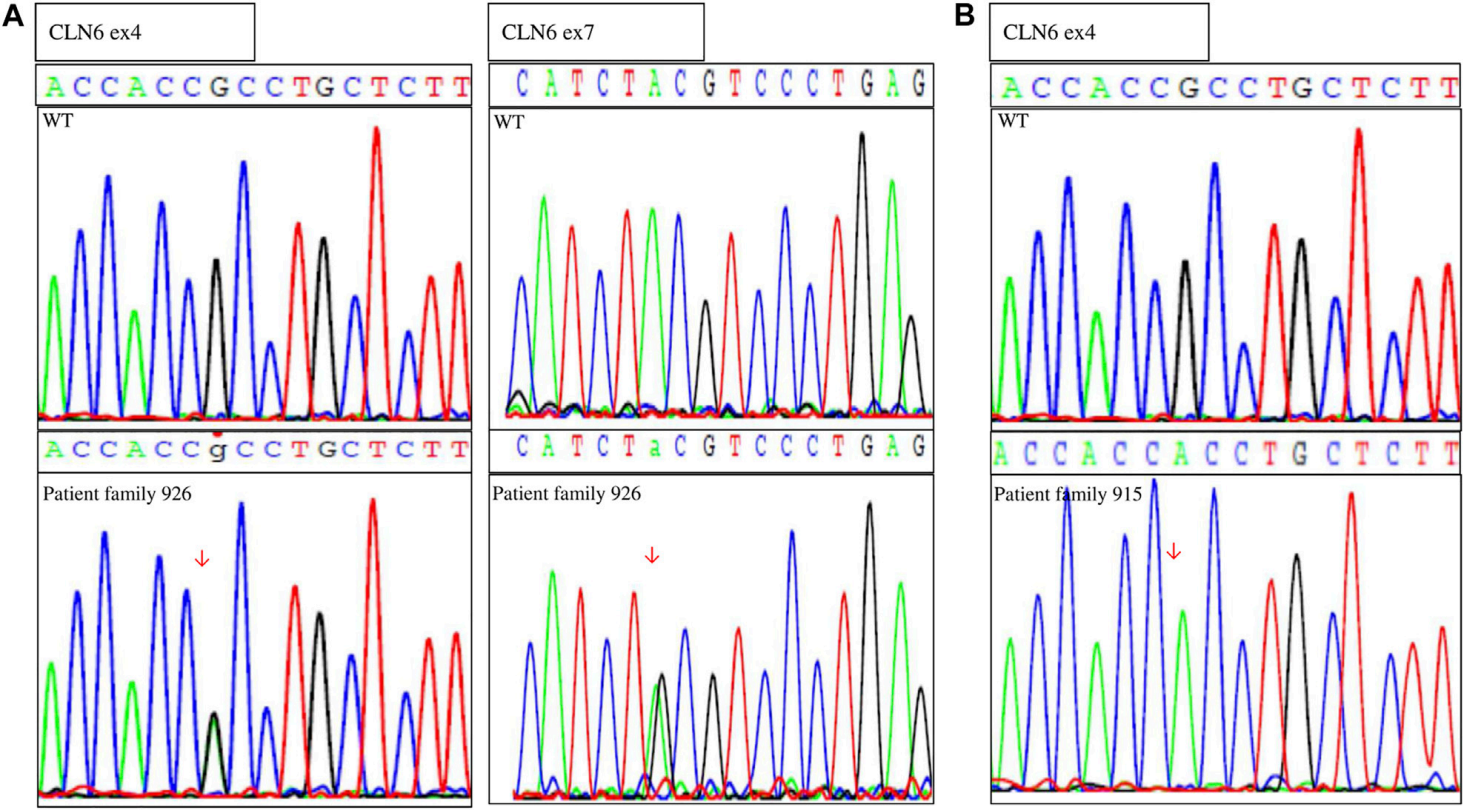
Sequences showing the **(A)** compound heterozygous variants in genomic DNA of the proband (family 926) and the wild-type (WT) individual and **(B)** homozygous variant of the proband (family 915) and the WT individual.

After identification of the *CLN6* homozygous variant by WES *in silico* panel analysis in family 915, Sanger sequence analysis of the *CLN6* exons and intron–exon boundaries of the proband of family 926 was performed. Molecular analysis revealed two *CLN6* variants: c.407G > A, p.Arg136His in exon 4 and c.884A > G, p.Tyr295Cys, in exon 7 (Figure 2). Segregation family analysis revealed that the c.407G > A variant was inherited from the mother. The c.884A > G variant appeared *de novo*. Microsatellite analysis (data not shown) excluded any possibility of non-paternity.

These variants (c.407G > A and c.884A > G) were absent from 300 chromosomes from Cypriot control samples.

### *In silico* Prediction Analysis and Structural Modeling of the *CLN6* Variants

To evaluate the relative importance of the novel variant, we carried out an *in silico* prediction of the effect of the amino acid changes on the protein using several prediction tools.

PolyPhen-2 analysis showed that variant p.Arg136His is predicted to be probably damaging with a score of 1.000 and 0.923 for HumDiv and HumVar, respectively. Variant p.Tyr295Cys is predicted also to be probably damaging with a score of 1.000 and 0.999 for HumDiv and HumVar, respectively (Table 3).

**TABLE 3:** PolyPhen-2, PROVEAN, SIFT, and MutationTaster prediction of the identified *CLN6* variants.

Variation Input	PolyPhen-2			PROVEAN		SIFT		Mutation taster	
	Prediction	SCORE HumDiv	SCORE HumVar	Prediction (cutoff = −2.5)	Score	Prediction (cutoff = 0.05)	Score	Prediction	Score
CLN6 136 R > H	Probably damaging deleterious disease causing	1	0.923	Damaging deleterious disease causing	−3.72	Damaging	0	Damaging deleterious disease causing	29
CLN6 295 Y > C	Probably damaging	1	0.999	Deleterious	−5.28	Damaging	0.021	Disease causing	194

PROVEAN and SIFT analyses showed that variants p.Arg136His and p.Tyr295Cys are predicted by PROVEAN to be damaging with scores of 3.72 and 5.28, respectively, and by SIFT to be damaging with scores of 0 and 0.021, respectively (Table 3).

MutationTaster analysis showed that both variants p.Arg136His and p.Tyr295Cys are predicted to be disease-causing (Table 3).

VarCards analysis revealed similar results with additional information as shown in Supplementary Table 1.

### Modeling Analysis

#### RaptorX and PyMOL

**FIGURE 3:**
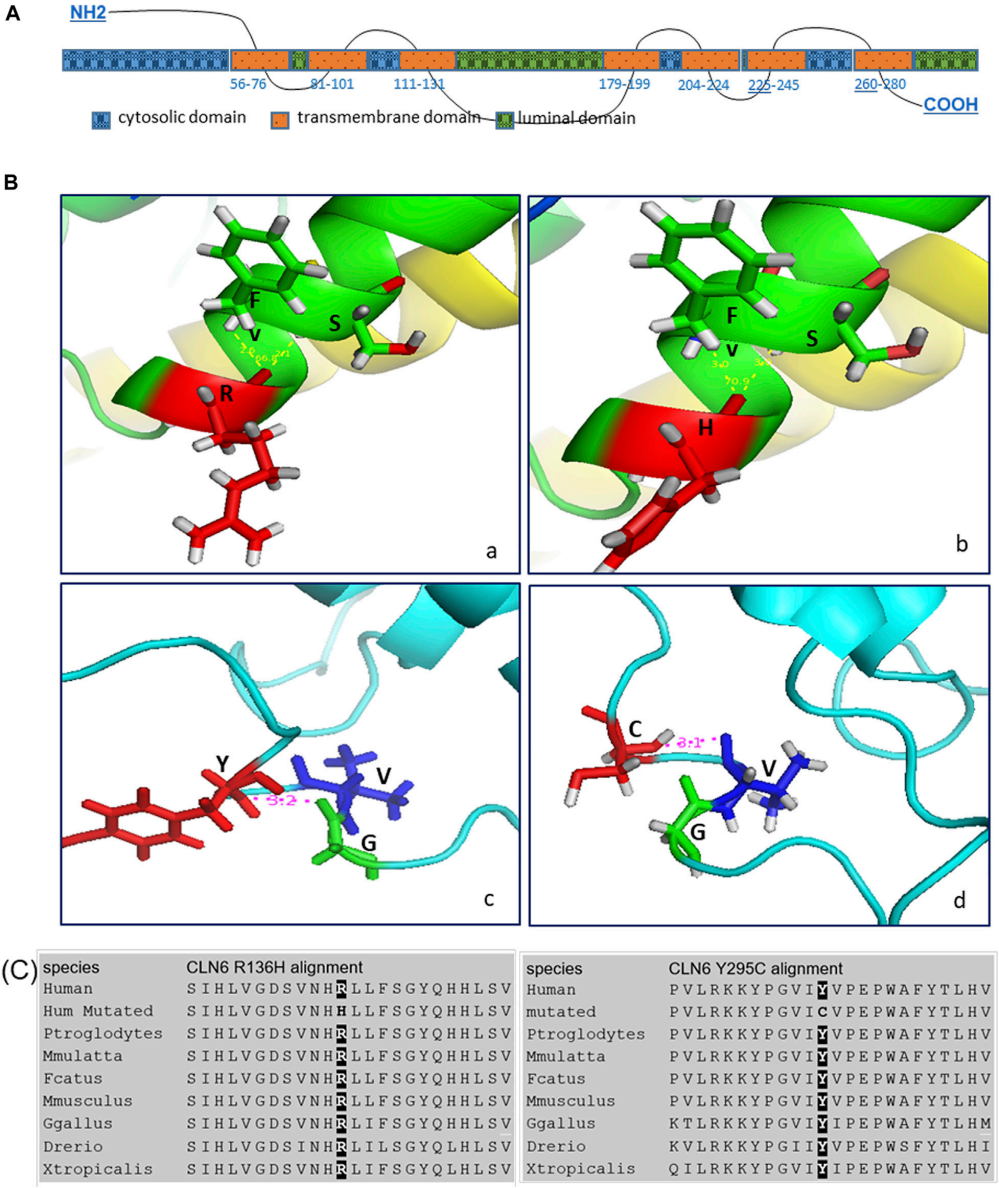
**(A)** CLN6 protein predicted domains. **(B)** *In silico* structural analysis showed the predicted CLN6 protein 3D structure, based on Protein Data Bank (PDB) ID: *2yevA*, *3wajA*, *5amrA*, *3ayfA*, *5i6xA*, and *3rkoB* of the wild-type arginine (a)/mutant histidine (b) at position 136 (shown in red color) and of the wild-type tyrosine (c)/mutant cysteine (d) at position 295 (shown in red color). The interacting molecules are shown in green and blue colors, and the structures are shown in the same scale and orientation. The corresponding numbers between the bonds are in angstrom (Å) units, and the angles are in degree (°). **(C)** Alignment analysis showed the arginine/histidine at position 136 and the tyrosine/cysteine at position 295 conservation among different species.

*In silico* structural analyses of the WT and variant CLN6 proteins provide a possible explanation of how the variants may affect the structure of the protein. More specifically, the p.Arg136His variant within the ER luminal domains–TM3–TM4 loop and variant p.Tyr295Cys at the ER luminal domains–C-terminus (Figure 3A) may affect the binding activity of these domains.

Arginine (WT) at position 136 creates two bonds, with phenylalanine at position 139 and serine at position 140. The distance of Arg-Phe is 3.0 Å and of Arg-Ser is also 3.0 Å. The angle Phe-Arg-Ser is 66.8°. Histidine (variant) also creates bonds with phenylalanine and serine, and the distance of His-Phe is 2.6 Å and of His-Ser is 2.1 Å. The angle Phe-His-Ser is 70.9°.

Furthermore, tyrosine (WT) at position 295 creates a bond with glycine at position 292, and the distance of Tyr-Gly is 3.2 Å; however, cysteine (variant) at position 295 creates a different bond with valine at position 293, and the distance of Cys-Val is 3.1 Å (Figure 3B).

In addition, protein sequence alignment showed that arginine at position 136 and tyrosine at position 295 are highly conserved (Figure 3C).

## Discussion

NCL or Batten disease is a rare, autosomal recessive, neurodegenerative disease usually of infantile-onset. The clinical signs typically appear at the ages of 3–8 years, starting with seizures and motor loss, followed by speech impairment, ataxia, cerebellar atrophy, myoclonus, and mental deterioration. NCLs reportedly account for approximately 20% of cerebellar atrophy cases in children and represent the second most common cause after mitochondrial disease worldwide (Chin et al., 2019; Matsumoto et al., 2019). However, NCLs are rare diseases, and through this report, we describe for the first time two unrelated Cypriot families with NCL due to biallelic *CLN6* variants.

The two Cypriot families harbored compound heterozygous variants (c.407G > A and c.884A > G) and a homozygous (c.407G > A) variant in *CLN6*.

However, the clinical phenotypes of the two families were different. The age of onset of the probands of the two families was the same, and the symptoms have started with motor impairment, followed by speech impairment, ataxia, and mental deterioration. Patients from both families did not show early signs of vision or hearing impairment. Furthermore, the proband of family 926 had rolandic epilepsy at the early stages of the disease, later on presenting with intractable myoclonic epileptic seizures, whereas the patients of family 915 did not show any signs of epilepsy. Epilepsy is one of the main symptoms of NCLs caused by *CLN6* variants; however, the patients of family 915 did not have epilepsy, and that is in agreement with some other studies. Karaca *et al.* reported patients with the same variant in exon 4 of the *CLN6* gene (Arg136His) in a consanguineous Turkish family, not mentioning either seizures or epilepsy. The clinical features were intellectual disability, cerebellar atrophy, ataxia, and cryptorchidism (no detailed clinical features were described in this report) (Karaca et al., 2015). Furthermore, Jain *et al.* in 2016 reported a consanguineous family with late-infantile NCL, without seizures and epilepsy. The proband had a homozygous missense *CLN6* variant (p.L270P) in exon 7, and he presented with loss of speech, gait abnormality with loss of ambulation, spasticity in the lower limbs, and distal dyskinesia; and brain MRI revealed mild cerebellar atrophy (Jain et al., 2016).

*CLN6* variants were described for the first time in 1997 to be associated with Batten disease (Sharp et al., 1997). Since then, more than 70 pathogenic variants have been identified, as described in the NCL mutation database (https://www.ucl.ac.uk/ncl-disease/mutation-and-patient-database/mutation-and-patient-datasheets-human-ncl-genes/cln6). In almost all the cases of NCLs caused by biallelic *CLN6* variants, visual loss is the main symptom; however, there are some reports without visual impairment. The first case was reported by Cannelli *et al.* in 2009, who described three families with no visual impairment. Another case report came from Sun *et al.* in 2018, who described a boy without visual issues. Furthermore, two unrelated patients without visual impairment were reported by Chin *et al.* in 2019 (Cannelli et al., 2009; Sun et al., 2018; Chin et al., 2019). These features agree with features present in the patients of our families.

The *CLN6* variant (c.407G > A, p.Arg136His) in exon 4 was previously reported in a homozygous state in a consanguineous Turkish family as described above (Karaca et al., 2015). Furthermore, Cannelli *et al.* described a compound heterozygous variant (c.406C > T, p.Arg136Cys and c.426C > G, p.Tyr142Ter) with one of these variants at the same amino acid position, arginine 136, change to cysteine instead of histidine (Cannelli et al., 2009). Variant c.407G > A results in a change of a highly conserved (down to frog *Xenopus tropicalis*), positively charged (basic, non-acidic amino acids, side chains often form salt bridges), polar, amphiphilic amino acid arginine at position 136 to a positively charged (basic, non-acidic amino acids), polar (form hydrogen bonds as proton donors or acceptors), and hydrophilic amino acid histidine (p.Arg136His). The *CLN6* variant (c.884A > G, p.Tyr295Cys) in exon 7 is a novel *de novo* variant that has not been reported previously. The c.884A > G *de novo* variant is present in this patient in combination with the p.Arg136His variant, consistent with biallelic inheritance. To our knowledge, this is the first report of a *de novo* variant in *CLN6*. The variant c.884A > G results in a change of a highly conserved (down to frog *X. tropicalis*), aromatic, nonpolar tyrosine to a polar, non-charged cysteine (p.Tyr295Cys) amino acid.

In this study, we also performed *in silico* prediction and structural analyses for the identified *CLN6* variants to better understand their effect on protein structure and function. *In silico* prediction analysis showed that both variants are damaging or probably damaging, deleterious, and disease-causing with a relatively high score. These results are in agreement with previous studies that showed variants in ER luminal domains at TM3–TM4 loop (p.Arg136Cys, p.Arg149Cys, p.Pro159Leu, p.Leu162Arg, p.Leu169Pro, and p.Tyr172Leu) and variants in ER luminal domains at the C-terminus (p.Pro297Thr, p.Glu298Lys, p.Pro299Leu, and p.Trp300Arg) to be pathogenic (Wheeler et al., 2002; Sun et al., 2018).

*In silico* structural analysis of these *CLN6* variants revealed a change in amino acid interaction and consequently in the 3D structure of the protein. Arginine at position 136 (WT amino acid) interacts with phenylalanine at position 139 and with serine at position 140. When mutated (Arg > His), the interactions with phenylalanine and serine are sustained; however, the distance from the histidine amino acid and the angle Phe-His-Ser is decreased. CLN6 has a cytosolic N-terminus, 7 TM domains, cytosolic and luminal loops that are connecting the TM domains, and a C-terminus in the ER lumen (Wheeler et al., 2002; Heine et al., 2007). These loops are all small (<15 amino acids), except the second luminal loop, which is longer, and it has 48 amino acids (Figure 3A). Bajaj *et al.* showed that the second luminal loop of CLN6 is required for the interaction of CLN6 with the lysosome enzymes. Therefore, the absence of CLN6 results in incompetent ER transfer of lysosomal enzymes and reduced levels of the enzymes at the lysosome (Bajaj et al., 2020). Tyrosine at position 295 interacts with glycine at position 292, and when mutated (Tyr > Cys), interaction with glycine is lost, and cysteine interacts with valine at position 293. In addition, Heine *et al.* indicated that the vesicular transport machinery in neuronal mouse cells fails to recognize the membrane region containing a C-terminally truncated CLN6 protein that is essential for proper targeting (Heine et al., 2007).

In conclusion, we present three patients from two unrelated families with clinical MRI and neurophysiology findings suggestive of JNCL. Both probands had biallelic variants in *CLN6*, confirming a diagnosis of atypical JNCL. Furthermore, *in silico* prediction analysis confirmed the likely pathogenicity of the two variants, and protein structural analysis displayed a possible explanation of how these variants may affect the binding activity and the function of the CLN6 protein and consequently cause the disease.

## Data Availability

The datasets presented in this study can be found in online repositories. The names of the repository/repositories and accession number(s) can be found below: https://www.ncbi.nlm.nih.gov/clinvar/docs/submit/, SCV001759935.
