# Supplementary material for: A Novel CLN6 Variant Associated With Juvenile Neuronal Ceroid Lipofuscinosis in Patients With Absence of Visual Loss as a Presenting Feature
Source: Front Genet. 2021 Nov 19;12:746101. doi: 10.3389/fgene.2021.746101 (PMC8640139; doi:10.3389/fgene.2021.746101)
Supplement: Supplementary file 1 [file Table1.DOCX]

Supplementary Table 1: VarCards in silico prediction of the CLN6 mutations

|  | **R136H** | | **Y295C** | |
| --- | --- | --- | --- | --- |
| **algorithm** | **score** | **prediction** | **score** | **prediction** |
| SIFT | 0 | Damaging | 0.021 | Damaging |
| Polyphen-2_HDIV | 1 | Probably_damaging | 1 | Probably_damaging |
| Polyphen-2_HVAR | 0.942 | Probably_damaging | 0.999 | Probably_damaging |
| LRT | 0 | Deleterious | 0 | Deleterious |
| MutationTaster | 1 | Disease_causing | 1 | Disease_causing |
| MutationAssessor | 2.16 | Medium | 2.28 | Medium |
| FATHMM | -3.95 | Damaging | -3.97 | Damaging |
| PROVEAN | -3.72 | Damaging | -5.28 | Damaging |
| VEST3 | 0.957 | Damaging | 0.952 | Damaging |
| MetaSVM | 1.056 | Damaging | 1.047 | Damaging |
| MetaLR | 0.919 | Damaging | 0.922 | Damaging |
| M-CAP | 0.547 | Damaging | 0.433 | Damaging |
| CADD | 35 | Damaging | 25.9 | Damaging |
| DANN | 1 | Damaging | 0.998 | Damaging |
| FATHMM_MKL | 0.952 | Damaging | 0.945 | Damaging |
| Eigen | 0.722 | Damaging | 0.703 | Damaging |
| GenoCanyon | 1 | Damaging | 1 | Damaging |
| fitCons | 0.722 | Damaging | 0.732 | Damaging |
| GERP++ | 5.14 | Conserved | 5.34 | Conserved |
| phyloP | 7.446 | Conserved | 7.674 | Conserved |
| phastCons | 1 | Conserved | 1 | Conserved |
| SiPhy | 18.623 | Conserved | 15.318 | Conserved |
| REVEL | 0.952 | Damaging | 0.958 | Damaging |
| ReVe | 0.949 | Damaging | 0.987 | Damaging |
| ClinPred | 0.905568 | pathogenic | 0.9968658 | pathogenic |
